# Up-regulation of angiotensin-converting enzyme in response to acute ischemic stroke via ERK/NF-κB pathway in spontaneously hypertensive rats

**DOI:** 10.18632/oncotarget.21156

**Published:** 2017-09-22

**Authors:** Zhou Ou, Meng-Xing Tao, Qing Gao, Xue-Ling Zhang, Yang Yang, Jun-Shan Zhou, Ying-Dong Zhang

**Affiliations:** ^1^ Department of Neurology, Nanjing First Hospital, Nanjing Medical University, Nanjing, People’s Republic of China; ^2^ Department of Neurology, Suqian City People’s Hospital, Suqian, People’s Republic of China

**Keywords:** renin-angiotensin system, angiotensin-converting enzyme, mitogen-activated protein kinase, nuclear factor-κB, acute ischemic stroke

## Abstract

Cerebral ischemic stroke is usually caused by a temporary or permanent decrease in blood supply to the brain. Despite general progress in diagnosis and treatment, the prognosis of stroke is still unsatisfactory, and more detailed potential mechanisms are needed to investigate underlying the pathological process. Here, we showed that serum angiotensin-converting enzyme (ACE) concentration was positively correlated with infarct volume after acute ischemic stroke (AIS). Moreover, using a permanent middle cerebral artery occlusion rat model, we indicated for the first time that increased ACE expression in response to AIS was regulated by the ERK/NF-κB pathway in peri-infarct regions. More importantly, we disclosed that angiotensin II type 1 receptors were implicated in up-regulation of ACE expression in peri-infarct regions. These findings offer insight into ACE expression and activity in response to stroke, and further our understanding of ACE mechanisms.

## INTRODUCTION

Acute cerebral infarction, the leading cause of death in the world, is usually caused by a temporary or permanent decrease in the blood supply to the brain [1, 2]. Despite general progress in diagnosis and treatment, the prognosis of stroke is still unsatisfactory and more precise potential mechanisms are needed to investigate underlying the pathological process.

Renin–angiotensin system (RAS) is now well accepted as a dual system, consisting of a circulating RAS and local RAS. Under physiological conditions, the components of circulating RAS cannot cross the blood–brain barrier (BBB), and local independent brain RAS participates in the pathophysiology of various disorders, including ischemic stroke [3, 4]. Angiotensin-converting enzyme (ACE) is an indispensable element of RAS that converts angiotensin I (Ang I) to angiotensin II (Ang II),and is present in cerebral microvessels of the rat [5]. Prior studies revealed that ACE was implicated in the pathological process of brain ischemic injury as well as cardiovascular disorders [6–8]. Furthermore, some clinical evidence showed that serum ACE activity increased after acute ischemic stroke (AIS), while blockade of ACE was effective in preventing recurrent stroke, beyond the effect of lowering blood pressure [9]. However, in contrast, a previous study from Catto and colleagues found that serum ACE activity were markedly reduced during the acute phase of cerebral infarction [10]. In this regard, these controversial findings lead to the challenge of whether ACE inhibitors can be used for anti-hypertension, and indeed, inhibit ACE activity in the days following AIS.

Mitogen-activated protein kinase (MAPK) cascade serves as a crucial intracellular mediator of responses closely related to cell growth and differentiation, stress, survival, and cell death [11]. The cascade is comprised of extracellular signal-regulated kinase (ERK), c-Jun N-terminal kinase (JNK), and p38 MAPK [12, 13]. Several lines of evidence have shown that ERK activation might decrease ACE gene expression and activate nuclear factor-κB (NF-κB). Meanwhile, recent research from our group found that blockage of NF-κB was involved in neuroprotection of angiotensin (Ang)-(1–7) in rats with permanent cerebral ischemia [14]. Additionally, a recent study from Meng and colleagues indicated that ACE2/Ang-(1–7)/Mas axis prevented lung fibrosis via repression of ERK/NF-κB signaling pathway [15]. On basis of above mentioned findings, we speculated that ERK/NF-κB signaling pathway contributed to modulation of RAS expression after cerebral ischemic stroke in peri-infarct regions. Although there were numerous clinical reports and even at times conflicting, animal data was limited about the serum ACE activity after AIS in the past. Therefore, in this study, we firstly enrolled patients with AIS to examine the relationship between serum ACE levels and infarct volume after AIS. Then we hypothesized that up-regulation of ACE in response to AIS may regulated by ERK/NF-κB signaling pathway via angiotensin II type 1 receptor (AT_1_R) in a spontaneously hypertensive rat.

## RESULTS

### Serum ACE levels are positively correlated with infarct volume after AIS

**Figure 1:**
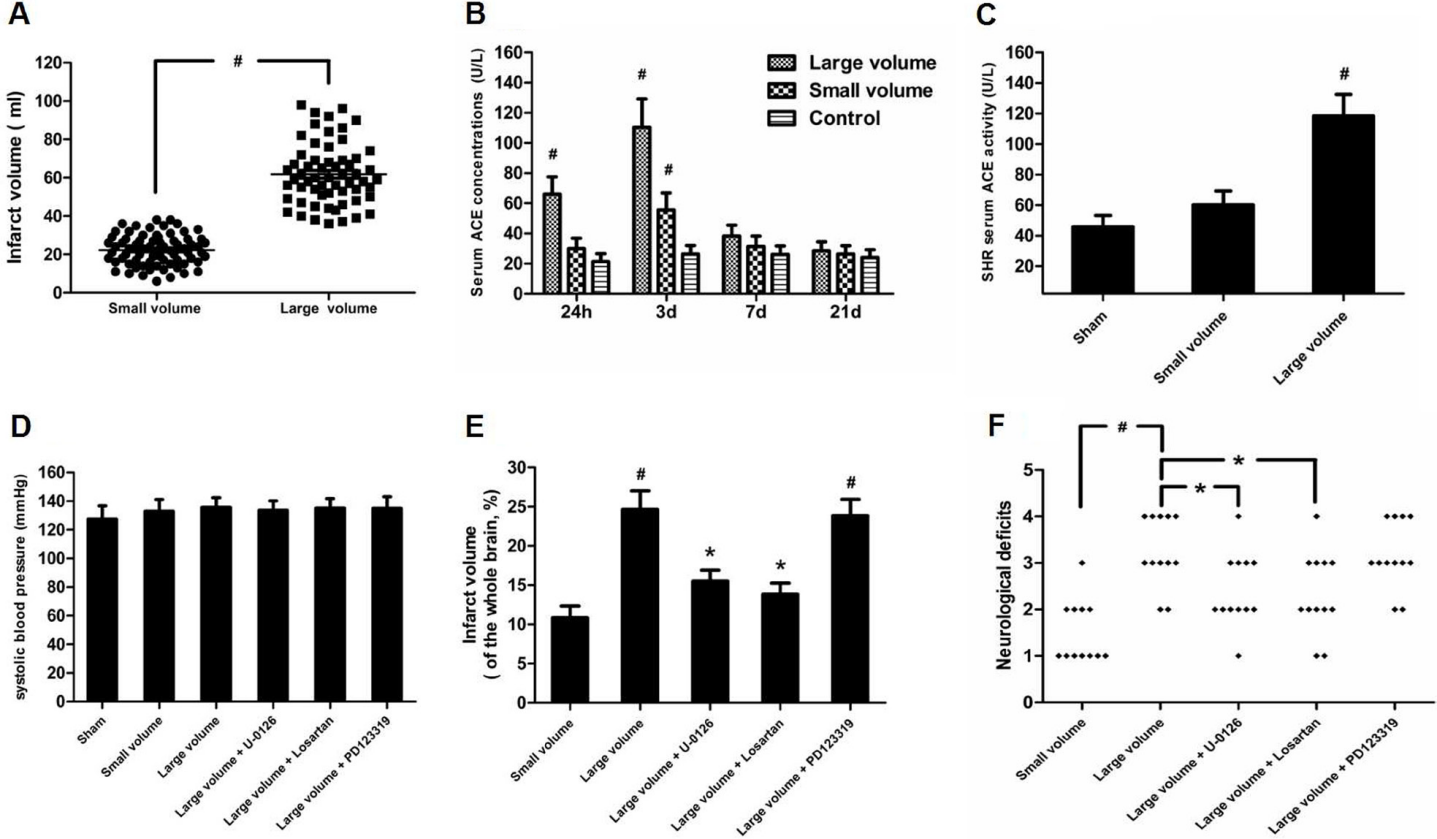
Effect of infarct volume on serum ACE concentration and neurological deficits in response to AIS/pMCAO (**A**) Infarct volume was measured from DWI scans using MIPAV software in patients with AIS. Data were analyzed by independent samples *t*-test. Columns represent mean ± SD. ^#^*P* < 0.05 vs. small volume group. (**B**) Serum ACE concentration in patients with AIS were examined by ELISA. Data were analyzed by one-way ANOVA followed by Tukey’s post-hoc test. Columns represent mean ± SD. ^#^*P* < 0.05 vs. control group. (**C**) Serum ACE concentrations in pMCAO rats were evaluated by ELISA (*n* = 6 per group). Data were analyzed by one-way ANOVA followed by Tukey’s post-hoc test. Columns represent mean ± SD. ^#^*P* < 0.05 vs. sham-operated group. (**D**) Systolic blood pressure was measured throughout the experiment using a tail cuff method (*n* = 6 per group). Data were analyzed by one-way ANOVA followed by Tukey’s post-hoc test. Columns represent mean ± SD. (**E**) Infarct volume was determined by TTC assay in pMCAO rats (*n* = 6 per group). Data were analyzed by one-way ANOVA followed by Tukey’s post-hoc test. Columns represent mean ± SD. ^#^*P* < 0.05 vs. small volume group. ^*^P < 0.05 vs. large volume group. (**F**) Neurological deficits were examined at 24 h after pMCAO based on a 5-point scale (*n* = 12 per group). Data were analyzed by Mann–Whitney *U*-test. ^#^*P* < 0.05 vs. small volume group. ^*^P < 0.05 vs. large volume group.

Firstly, we examined infarct volume in a large infarct volume group (middle cerebral artery [MCA] stem occlusion) and small infarct volume group (occlusion of MCA distal branches). As expected, we found that the large volume group exhibited markedly higher infarct volume than small infarct volume group (*P* < 0.05) (Figure 1A).Next, we measured serum ACE levels in AIS patients and controls. As shown in Figure 1B, when compared to control subjects, serum ACE levels were significantly increased in the large volume group within 24 h and 3 days after stroke by approximately 3.1-fold (*P* < 0.05) and 4.2-fold (*P* < 0.05), respectively. Similarly, serum ACE concentrations increased in the small volume group within 24 h and 3 days after stroke by approximately 32% (*P* > 0.05) and 2.1-fold (*P* < 0.05), respectively. Further, serum ACE levels were restored to normal levels in both the large volume and small volume groups, similar to control subjects, at 7 days after AIS, indicating that serum ACE levels were closely correlated with infarct volume. We also obtained serum ACE levels from tail vein blood of animals before they were sacrificed, at 24 h after permanent MCA occlusion (pMCAO). Compared to sham-operated rats, serum ACE levels showed more significant increase in the large infarct volume group (*P* < 0.05) than that in the small infarct volume group (*P* > 0.05) (Figure 1C). This confirms that serum ACE levels are closely related with infarct volume after AIS. National Institute of Health Stroke Scale (NIHSS) scores was used to assess the severity of cerebral damage in each person at admission. We found that the significantly increased serum ACE concentration within 3 days after an acute event was strongly associated with more NIHSS score (*P* < 0.05). Taken together, these data suggest that serum ACE levels are positively correlated with infarct volume after AIS.

### ACE expression in response to AIS is increased via ERK/NF-κB pathway

**Figure 2:**
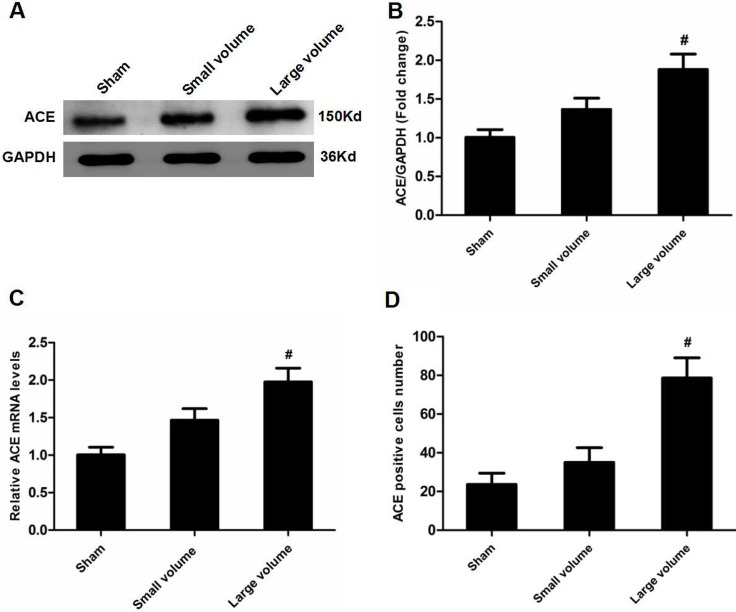
Effect of infarct volume on ACE expression in peri-infarct regions at 24 h after pMCAO (**A**) ACE protein levels were examined by western blot assay (*n* = 6 per group). (**B**) Quantitative analysis of ACE protein levels (*n* = 6 per group). (**C**) Quantitative analysis of ACE mRNA levels (*n* = 6 per group). (**D**) Quantitative analysis of ACE-positive cell number (*n* = 6 per group). Data were analyzed by one-way ANOVA followed by Tukey’s post-hoc test. Columns represent mean ± SD. ^#^*P* < 0.05 vs. sham-operated group.

**Figure 3:**
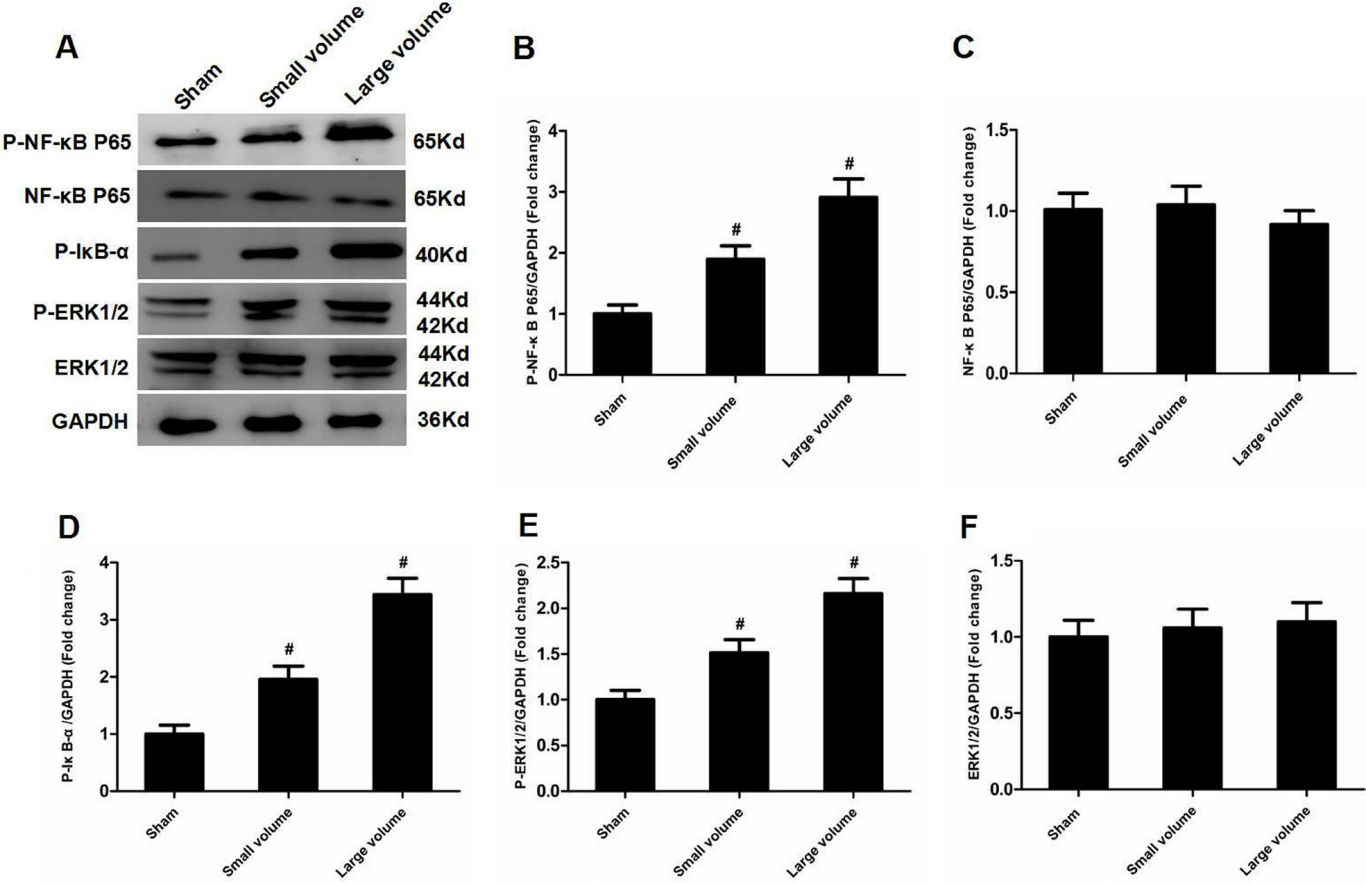
Effect of infarct volume on activation of ERK and NF-κB in peri-infarct regions at 24 h after pMCAO (**A**) Protein levels of p-NF-κB p65, p-NF-κB p65, p-IκB-α, p-ERK1/2, and ERK1/2 were examined by western blot analysis (*n* = 6 per group). (**B**) Quantitative analysis of p-NF-κB p65 protein levels (*n* = 6 per group). (**C**) Quantitative analysis of NF-κB p65 protein levels (*n* = 6 per group). (**D**) Quantitative analysis of p-IκB-α protein level (*n* = 6 per group). (**E**) Quantitative analysis of p-ERK1/2 protein levels (*n* = 6 per group). (**F**) Quantitative analysis of ERK1/2 protein levels (*n* = 6 per group). Data were analyzed by one-way ANOVA followed by Tukey’s post hoc test. Columns represent mean ± SD. #*P <* 0.05 vs. sham-operated group.

As seen in Figure 1D, there was no significant difference in blood pressure levels among groups after anti-hypertension induced by amlodipine (10 mg/kg/d). In the large volume group, pMCAO led to a wide range of infarction in the cerebral cortex and subcortical regions, which manifested as evident neurological deficits. As compared to small volume group, the large volume group exhibited higher infarct volumes (24.6 ± 2.8 vs. 11.8 ± 1.7% of whole brain, *n* = 6, *P* < 0.05) and neurological deficits (median of large volume group: 3, *n* = 12, *P* < 0.05) (Figure 1E, 1F). To examine ACE mRNA and protein expression in the peri-infarct area and corresponding area of sham-operated rats, RT-PCR and western blot assays were performed. Compared with sham-operated rats, ACE mRNA levels were markedly increased in the large volume and small volume groups after 24 h of pMCAO by approximately 2.0-fold (*P* < 0.05) and 45% (*P* > 0.05), respectively (Figure 2C). Similar data were obtained for ACE protein levels (Figure 2A, 2B). Afterwards, we further employed immunohistochemistry staining to assess ACE expression in peri-infarct area after pMCAO. Small and large infarct volumes increased the number of ACE-positive neurons by approximately 37% (*P* > 0.05) and 2.3-fold (*P* < 0.05), respectively (Figure 2D).Furthermore, we used western blot assays to examine protein levels of NF-κB p65, phosphorylated form of NF-κB p65 (p-NF-κB p65), phosphorylated form of inhibitor of κB-α (p-IκB-α), ERK1/2 and phosphorylated form of ERK1/2 (p-ERK1/2). When compared with the sham-operated group, protein levels of p-NF-κB p65, p-IκB-α, and p-ERK1/2 were significantly increased in response to acute stroke after pMCAO (Figure 3A, 3B, 3D, 3E). In contrast, the protein levels of NF-κB p65 and ERK1/2 showed slight variations after stroke (Figure 3A, 3C, 3F).

**Figure 4:**
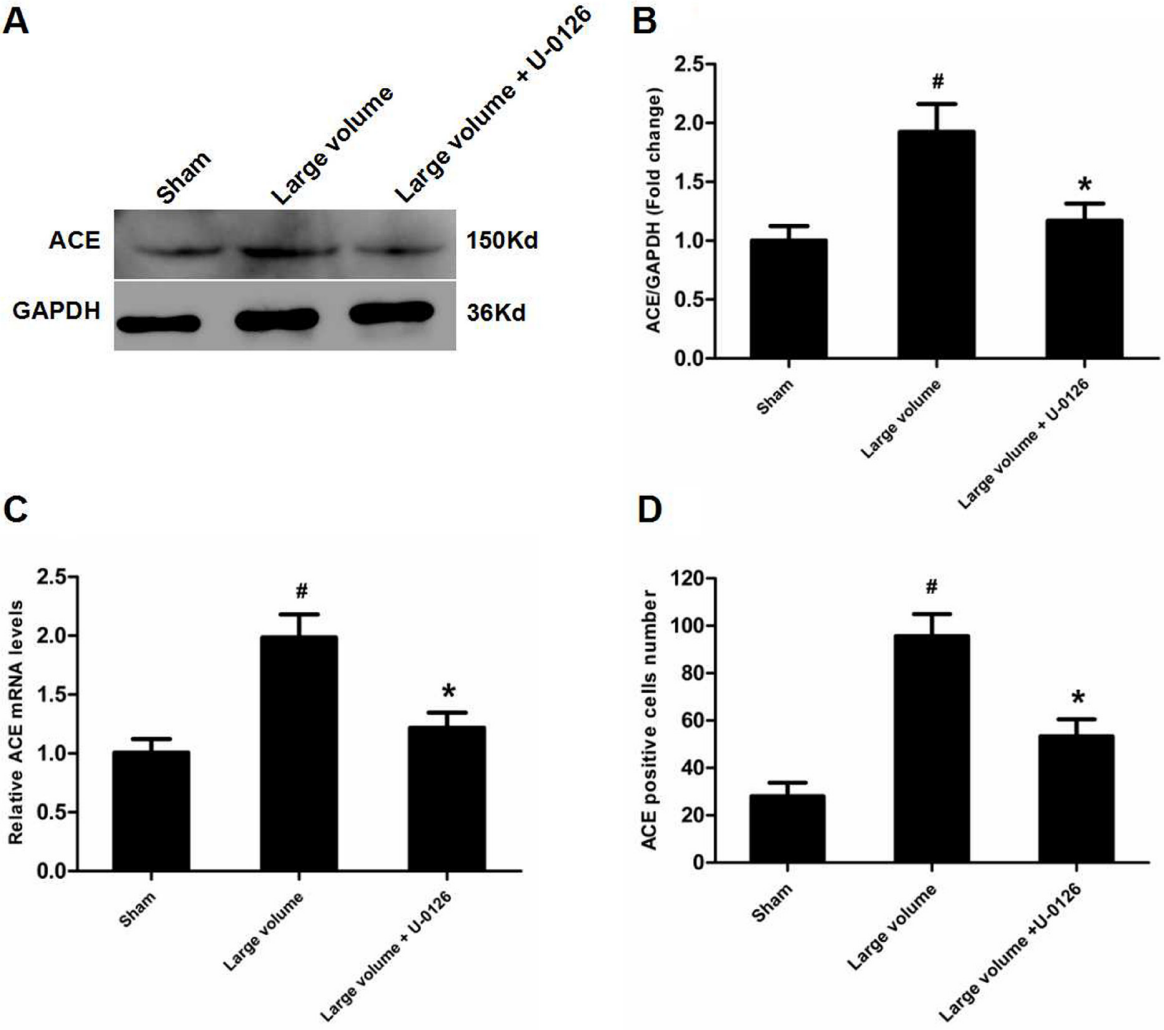
Inhibition of ERK contributes to down-regulation of ACE expression in peri-infarct regions induced by pMCAO (**A**) ACE protein levels were examined by western blot assay (*n* = 6 per group). (**B**) Quantitative analysis of ACE protein levels (*n* = 6 per group). (**C**) Quantitative analysis of ACE mRNA levels (*n* = 6 per group). (**D**) Quantitative analysis of ACE-positive cell number (*n* = 6 per group). Data were analyzed by one-way ANOVA followed by Tukey’s post hoc test. Columns represent mean ± SD. ^#^*P* < 0.05 vs. sham-operated group. ^*^P < 0.05 vs. large volume group.

**Figure 5:**
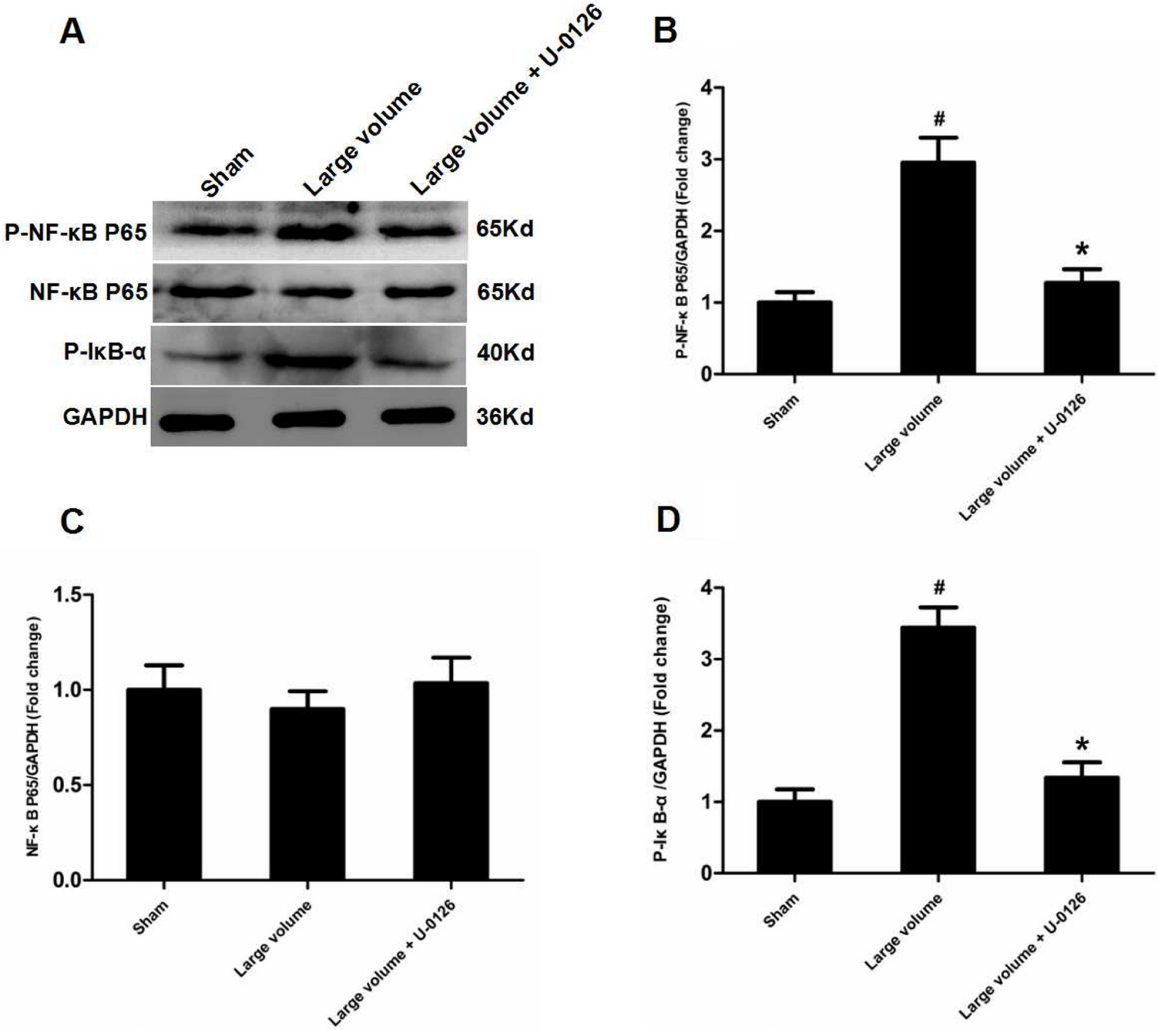
Inhibition of ERK is involved in deactivation of NF-κB in peri-infarct regions induced by pMCAO (**A**) Protein levels of p-NF-κB p65, p-NF-κB p65, and p-IκB-α were examined by western blot analysis (*n* = 6 per group). (**B**) Quantitative analysis of p-NF-κB p65 protein levels (*n* = 6 per group). (**C**) Quantitative analysis of NF-κB p65 protein levels (*n* = 6 per group). (**D**) Quantitative analysis of p-IκB-α protein levels (*n* = 6 per group). Data were analyzed by one-way ANOVA followed by Tukey’s post hoc test. Columns represent mean ± SD. ^#^*P* < 0.05 vs. sham-operated group. ^*^P < 0.05 vs. large volume group.

To further determine whether ERK/NF-κB pathway was involved in the modulation of ACE expression after pMCAO, we used U-0126 to inhibit ERK1/2. U-0126 significantly attenuated infarct volumes (24.6 ± 2.8 vs. 16.4 ± 1.6% of whole brain, *n* = 6, *P* < 0.05) and reversed the marked increase in neurological deficits (median of large volume + U-0126 group: 2, *n* = 12, *P* < 0.05) compared with the large volume group (Figure 1E, 1F). Additionally, increased ACE mRNA and protein levels induced by AIS were attenuated by U-0126 (Figure 4A–4C). Similarly, U-0126 abolished the increase in protein levels of p-NF-κB p65, p-IκB-α, and p-ERK1/2 caused by acute stroke (Figure 5A, 5B, 5D). However, protein levels of NF-κB p65 were not significantly altered (Figure 5A, 5C). Finally, increased number of ACE-positive neurons in the small and large infarct volume groups were mostly reversed by U-0126 (Figure 4D). Altogether, these results show that ERK/NF-κB pathway contributes to the up-regulation of ACE expression in response to AIS.

### AT_1_R are implicated in ACE up-regulation in response to AIS

**Figure 6:**
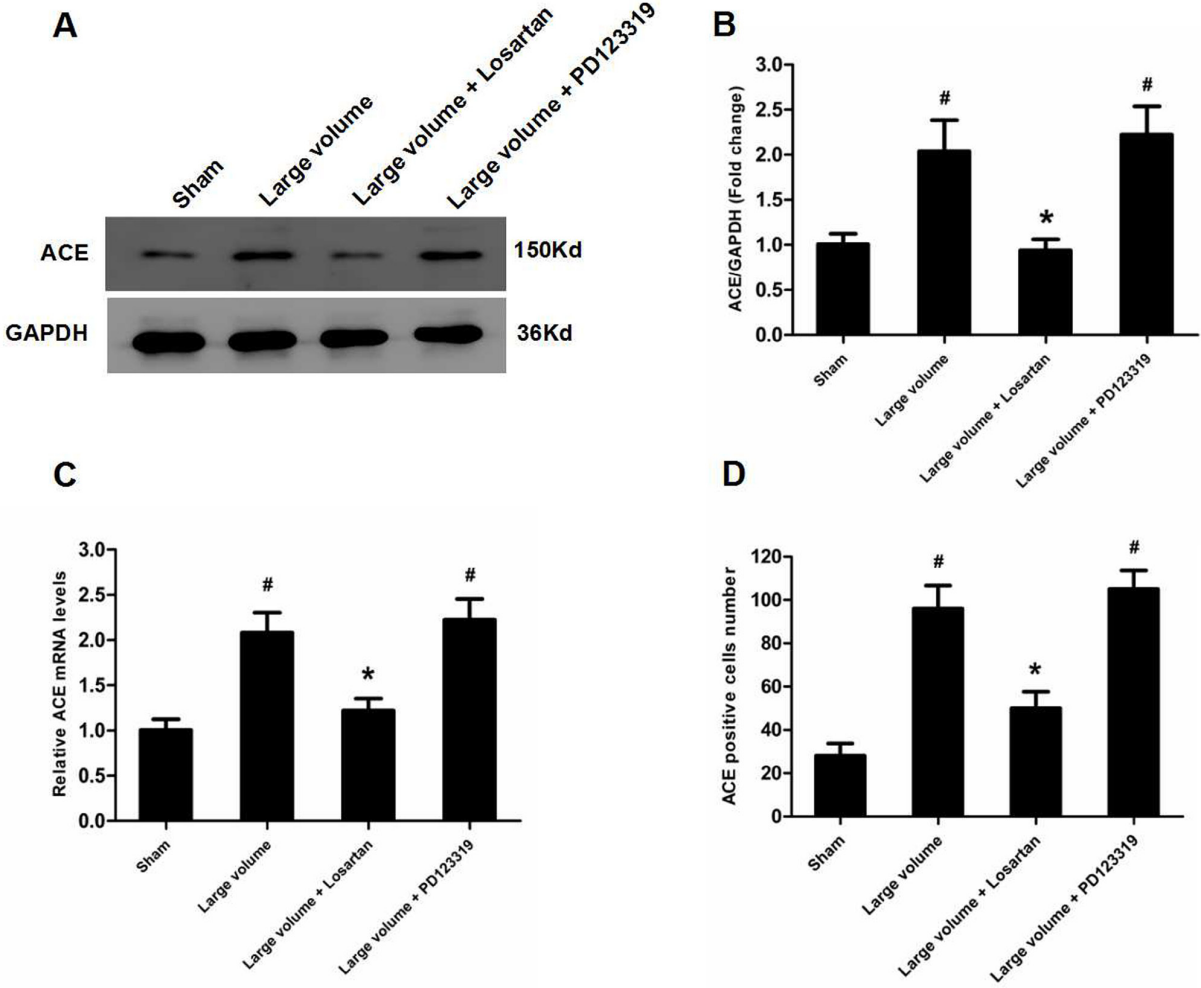
Up-regulation of ACE expression induced by pMCAO is mediated by AT_1_R rather than AT_2_R (**A**) ACE protein levels were examined by western blot assay (*n* = 6 per group). (**B**) Quantitative analysis of ACE protein levels (*n* = 6 per group). (**C**) Quantitative analysis of ACE mRNA levels (*n* = 6 per group). (**D**) Quantitative analysis of ACE-positive cell number (*n* = 6 per group). Data were analyzed by one-way ANOVA followed by Tukey’s post hoc test. Columns represent mean ± SD. ^#^*P* < 0.05 vs. sham-operated group. ^*^P < 0.05 vs. large volume group.

To confirm which type of Ang II receptors participated in the modulation of ACE, we pre-treated rats with inhibitors for AT_1_R (losartan) or AT_2_R (PD123319) 7 days before pMCAO. Compared with the large volume group, oral administration of losartan for 7 days significantly reduced the infarct volume (24.6 ± 2.8 vs. 14.8 ± 1.5% of whole brain, *n* = 6, *P* < 0.05) and attenuated the increase in neurological deficits (median of large volume + losartan group: 2, *n* = 12, *P* < 0.05) (Figure 1E, 1F). Moreover, increased ACE mRNA and protein levels after pMCAO were mostly reversed by losartan (Figure 6A–6C). Consistently, losartan ameliorated the increase in ACE-positive neuron number induced by acute cerebral infarction in pMCAO model rats (Figure 6D). In contrast, there was no remarkable effect of PD123319 on ACE expression, and altered ACE mRNA and protein levels triggered by AIS were unaffected (Figure 6A–6C). Additionally, no marked influence of PD123319 on ACE-positive neuron number in infarct areas was observed (Figure 6D). Thus, these findings reveal that AT_1_R are implicated in ACE up-regulation in response to AIS.

## DISCUSSION

ACE is the rate-limiting enzyme of RAS, and regulates conversion of inactive Ang I to active Ang II, thereby participating in pathogenesis of several neurological disorders including cerebral infarction [16]. Although numerous clinical researches have done about the relationship between ACE and AIS, no findings to date have shown whether serum ACE concentration and infarct volume after AIS are directly associated. Herein, we show for the first time that increased serum ACE levels and ACE expression in peri-infarct regions are positively correlated with infarct volume in patients with AIS, as well as a pMCAO rat model. These findings further support the application of ACE antagonists to inhibit ACE activity as well as anti-hypertension in the days following AIS.

There is wide agreement that the NF-κB family mainly consists of p65 (RelA) and p50/p105 (NF-κB1) subunits, which exist as cytoplasmic homo- or heterodimers, and are maintained in an inactive state by binding to IκB protein. When stimulated, IκB-a is firstly phosphorylated and then degraded by the 26S proteasome, which facilitates translocation of p50/p65 heterodimers into the nucleus to control gene transcription [17]. Prior studies indicated that NF-κB acted downstream of ERK in modulation of ACE2 expression in human primary retinal pigment epithelia cells [18]. Meanwhile, recent research from Garcia and colleagues was the first to demonstrate that NF-κB was a transcriptional factor for ACE, and ACE was activated via an ERK/NF-κB-dependent pathway in human microvascular endothelial cells [19]. According to above-mentioned findings, we hypothesized that ERK/NF-κB signaling pathway contributed to the up-regulaiton of ACE expression in response to AIS. Appropriately, our findings show that ERK and NF-κB expression are up-regulated along with increased ACE mRNA and protein levels. To further verify this hypothesis, we used an ERK inhibitor. As predicted, inhibition of ERK blocked increased ACE mRNA and protein levels, as well as ACE-positive neuron number and NF-κB expression. This was consistent with a previous study from Li and colleagues showed that the ERK/NF-κB pathway contributed to modulation of ACE2 expression in animal models of acute lung injury [20].

Considering that the type of Ang II receptors involved in mediation of ACE expression after ischemic stroke has rarely been demonstrated before, we investigated which type of Ang II receptors are involved in ACE activation in our study. Previous research has shown that AT_1_R are involved in the increase of ACE expression in a neuronal cell line [21]. In our study, we found that after AIS, increased ACE mRNA and protein levels, as well as ACE-positive neuron number, were completely abolished by losartan, an AT_1_R inhibitor. In contrast, an AT_2_R inhibitor, PD123319, had no influence on these actions in response to acute cerebral infarction. Thus, we confirm that up-regulation of ACE in response to AIS is dependent on AT_1_R, in this scenario at least. This result was further supported by a previous study from wang and colleagues revealed that scutellarin protected against cerebral ischemic injury via down-regulation of ACE in an AT_1_ receptor-dependent manner [16]. Additionally, we also revealed that pre-treated the rats with AT_1_R inhibitors can significantly reduce the infarct volume along with the decline of neurological deficits. These results need to be further verified whether pre-treat the AIS patients with AT_1_R antagonist could decrease the infarct volume and NIHSS score as compared with other kinds of anti-hypertension drugs in our future work [22–26].

In summary, we show that serum ACE concentration is positively correlated with infarct volume in AIS patients, as well as a pMCAO rat model. Moreover, we suggest that the ERK/NF-κB signaling pathway contributes to up-regulation of ACE expression in response to AIS in peri-infarct regions. More importantly, we reveal that up-regulation of ACE expression is dependent on AT_1_R. These findings offer insight into ACE expression and activity in response to stroke, and further our understanding of ACE mechanisms.

## MATERIALS AND METHODS

### Participants

Patients were examined in the Department of Neurology of Nanjing First Hospital and Suqian City People’s Hospital between October 2016 and January 2017. An informed consent was acquired from each individual. Approval of the experimental protocol was given by the ethics committees of Nanjing Medical University. At admission, baseline information of all patients was collected (Table 1) and all patients with AIS received a multiparametric magnetic resonance imaging (MRI) protocol that included diffusion weighted imaging (DWI) and magnetic resonance angiography (MRA). Patients were classified into two clinical categories depending on whether acute MCA main stem occlusion identified by MRA: large infarct volume (MCA stem occlusion, *n* = 60) or small infarct volume (occlusion of MCA distal branches, *n* = 72). Meanwhile, the infarct volume was evaluated basing on DWI. Serum samples were collected from patients at four time points: within 24 h, 3 days, 7 days and 21 days later after stroke onset. NIHSS scores were used to assess severity of cerebral damage at admission. The control group (*n* = 110) contained subjects with no history of stroke and normal MRI brain scans but who were referred to the neurology outpatient clinic for reasons unrelated to stroke. Inclusion criteria were as follows: (1) patients of all ages with AIS; (2) from onset of symptoms to blood sample collection < 24 h (time window < 24 h); and (3) ability to undergo MRI examination. Exclusion criteria were as follows: (1) patients with history of ACE inhibitor drug administration; (2) previous history of cerebrovascular events; (3) received intravenous thrombolytic therapy or interventional therapy; (4) severe heart, lung, liver, and renal dysfunction; and (5) inability to undergo MRI examination.

**Table 1:** Baseline demographics and clinical characteristics of the study population

	Control	Small volume	Large volume
No.patients (male/female)	110 (58/52)	72 (38/34)	60 (32/28)
Age, mean ± SD (years)	64.5 ± 13.7	68.2 ± 12.6	66.8 ± 14.2
Systolic blood pressure (mmHg)	156.4 ± 24.5	158.7 ± 23.6	162.9 ± 28.8
Diastolic blood pressure (mmHg)	86.5 ± 18.3	89.7 ± 16.6	92.1 ± 15.9
Heart rate (/min)	78.2 ± 11.4	82.5 ± 13.8	80.6 ± 14.5
Total cholesterol (mg/dL)	187.8 ± 38.6	179.2 ± 35.7	184.5 ± 40.4
Postprandial blood glucose (mg/dL)	132.9 ± 32.3	135.6 ± 30.4	142.5 ± 35.7
NIHSS score		9.5 ± 3.3	22.4 ± 9.2^#^

^#^*P* < 0.05 versus small volume. NIHSS, National Institute of Health Stroke Scale.

### Reagents

Antibodies against NF-κB p65, p-NF-κB p65, p-IκB-α, ERK1/2, p-ERK1/2, and β-actin (all used at 1:1000) were acquired from Cell Signaling Technology (Beverly, MA, USA). An antibody against ACE was provided by Abcam (ab77990; Abcam Inc., Eugene, OR, USA). Losartan (AT_1_R inhibitor), PD123319 (AT_2_R inhibitor), U-0126 (ERK1/2 antagonist), amlodipine (calcium ion antagonist), and 2,3,5-triphenyltetrazolium chloride (TTC) were obtained from Sigma Chemical Co. (St. Louis, MO, USA).

### Animals

Male spontaneously hypertensive rats (15–16 months of age, 400–450 g) were provided by the Experimental Animals Center of Nanjing Medical University. The rats were housed in an air-conditioned room under 12 h light and dark cycles. They were supplied with water and standard mouse diet. The protocol was approved by the Biological Research Ethics Committee of Nanjing First Hospital.

### Experimental groups and drug administration

All rats were allocated to six groups randomly (6 rats per group): sham, small infarct volume (infarct volume < 15% of whole brain), large infarct volume (infarct volume ≥ 15% of the whole brain), large infarct volume + U-0126, large infarct volume + losartan, and large infarct volume + PD123319. All groups were given daily oral administration of amlodipine (10 mg/kg/d) for 2 weeks before the start of the experiment. Rats in the large infarct volume + U-0126 group, large infarct volume + losartan group, and large infarct volume + PD123319 group received treatment with U-0126 (30 mg/kg intraperitoneally), losartan (50 mg/d per rat for oral administration), or PD123319 (50 mg/d per rat for oral administration), respectively, for one week before the onset of the experiment.

### Blood pressure measurement

Systolic blood pressure was measured throughout the experiment using a tail cuff method (BP-2000; Visitech Systems, Inc., Apex, NC, USA) [27]. All animals were subjected to this procedure with daily measurements and recordings for 2 weeks before the beginning of the experiment.

### pMCAO

Permanent middle cerebral artery occlusion was induced using an intraluminal filament, based on the methods of Longa [28]. Animals were firstly anaesthetized with 10% chloral hydrate (350 mg/kg, i.p.). Then, through a ventral midline incision, the right common carotid artery (CCA), external carotid artery (ECA), and internal carotid artery (ICA) were exposed and carefully isolated from adjacent nerves. Next, a 3.0-cm-length of filament (Φ 0.26 mm) was inserted from the right ECA into the ICA lumen, and advanced until there was resistance (1.8–2.0 cm from the bifurcation). Decreased cerebral blood flow (CBF) was validated using a laser Doppler flowmeter (moorVMS-LDF-1; Moor Instruments Inc., Axminster, UK). The model was deemed successful if there was > 80% decline in CBF after occlusion. The filament was kept in place until the rat was euthanized. During surgery, body temperature was maintained in the range of 37.0 ± 0.5°C with a heating pad and warm light. Rats in the sham-operated group received filament insertion into the ICA but with no drop in CBF.

### Determination of neurological deficits

An investigator who was blinded to the experimental groups evaluated neurological deficits at 24 h after pMCAO. Neurological results were based on a 5-point scale [29]: no neurological deficit = 0, failure to extend right paw fully = 1, circling to right = 2, falling to right = 3, and no spontaneous movement and depressed levels of consciousness = 4.

### Measurement of infarct volume

Rats were killed after 24 h of pMCAO. The brains were immediately removed and cut into five 3 mm-thick coronal sections. Sections were immersed in 1% TTC at 37°C for 30 min in the dark, and stained sections fixed with 4% paraformaldehyde. Normal brain tissue is dyed bright red, with the unstained area identified as the region of ischemic injury [30]. Infarct volumes were measured using the Image Pro-Plus 5.1 analysis system (Media Cybernetics Inc., Silver Spring, MD, USA), with Swanson’s method to rectify for edema [31]. Medical Image Processing, Analysis, and Visualization (MIPAV) software (version 3.0; National Institutes of Health [NIH], Bethesda, MD, USA) was utilized to measure infarct volumes in AIS patients identified by DWI [32].

### Enzyme-linked immunosorbent assay

Tail vein blood were collected from the rats before euthanized at 24 h after pMCAO. Subsequently, blood samples from AIS patients and pMCAO rats were employed to evaluate serum ACE concentrations (U/L) using an ELISA kits (R&D Systems Inc., Minneapolis, MN, USA) following the supplier’s guidelines [33]. In brief, samples were centrifuged at 1000 × g for 15 min at 4°C to remove cellular debris. Then the supernatant was collected to measure serum ACE concentrations. The absorbance at 450 nm in every well was evaluated with the spectrophotometer.

### Real-time PCR analysis

To quantify mRNA levels of related target genes, ACE mRNA expression was examined, as described previously [34]. In brief, total RNA was extracted from peri-infarct tissue and corresponding tissue of sham-operated rats using TRIzol (Invitrogen, Waltham, MA, USA). PrimeScript^™^ RT Master Mix (Takara Bio Inc., Japan) was used to reverse transcribe an equal quantity of total RNA under standard conditions. Afterwards, quantitative real-time PCR was performed using SYBR^®^ Premix Ex Taq^™^ (Takara Bio Inc.), with specific primers to detect ACE mRNA expression. Meanwhile, GAPDH was adopt as an internal control. Primers for target genes are listed as follows: ACE forward 5′-CTGGAGACCACTCCCATCCTTCTC-3′ and ACE reverse 5′-GATGTGGCCATCACATTCGTCA GAT-3′. GAPDH forward 5′-CGTCCCGTAGACAAAATG-3′, GAPDH reverse 5′-TAGTGGGGTCT CGCTCC-3′.

### Western blot analysis

Western blot assay was performed according to manufacture’s instructions [35]. Briefly, the peri-infarct regions and corresponding area of sham-operated rats was homogenized in lysis buffer (Beyotime Inc.). Following that step, the supernatants were collected as protein sample and protein concentrations were assessed by a BCA kit (Pierce, IL). Protein samples were separated on SDS Ready Gel Precast Gels (Bio-Rad, USA), transferred to a polyvinylidene difluoride membranes, and blocked with 5% non-fat dried milk for 2 h at room temperature. After that, the membranes were exposed to primary antibodies over night at 4°C and then incubated with the corresponding horseradish peroxidase (HRP)-coupled secondary antibodies for 2 h at room temperature. Finally, protein bands were visualized with the chemiluminescent HRP substrate (SuperSignal West Pico, Thermo Scientific Inc.) for 5min at room temperature, before exposure to X-ray film (Fujifilm Inc.). Band intensity was quantified using Quantity One software 4.6.2 (Bio-Rad Laboratories Inc.) and normalized to GAPDH.

### Immunohistochemistry analysis

Rats were killed and the brains were obtained and immersed in 4% paraformaldehyde over night. Coronal sections were provided by a sledge microtome [35]. Then the sections were dewaxed, hydrated and treated with 0.3% H_2_O_2_ for 30 min to quench the endogenous peroxidases. Afterwards, the slides were dealt with 0.5% Triton-X 100 for 30 min, blocked with 5% bovine serum albumin for 30 min, and exposed to primary antibodies over night at 4°C: monoclonal mouse anti-ACE (1:100, ab77990; Abcam Inc., USA). After that, the sections were incubated with secondary peroxidase conjugated goat anti-mouse IgG (1:1000, Cell Signaling Technology Inc.) for 1 h. Finally, the sections were counterstained with Mayer’s hematoxylin, dehydrated, and mounted on slides following the diaminobenzidine reaction. Cells with positive ACE immunoreactivity (blue granules) were counted as ACE-positive neurons. The microscope was equipped with a CCD camera to count cell number. Three independent investigators who were blinded to the experimental groups performed the counts.

### Statistical analysis

Statistical analysis was implemented by the SPSS software (version 17.0, SPSS). Statistical differences were assessed by an independent sample *t*-test and one-way ANOVA followed by Tukey’s post hoc test. Pearson correlation was used to analyze relationship of serum ACE concentration to NIHSS scores. Mann–Whitney *U*-test was employed to evaluate statistical differences of neurological deficits between groups. Data are presented as mean±SD except the neurological deficit. Differences were considered significant at *P* < 0.05.
